# Association between glaucoma surgery and all-cause and cause-specific mortality among elderly patients with glaucoma: a nationwide population-based cohort study

**DOI:** 10.1038/s41598-021-96063-7

**Published:** 2021-08-23

**Authors:** Sang Yeop Lee, Hun Lee, Ji Sung Lee, Sol Ah Han, Yoon Jeon Kim, Jae Yong Kim, Hungwon Tchah

**Affiliations:** 1 Department of Ophthalmology, Yongin Severance Hospital, Yonsei University College of Medicine, Yongin, Gyeonggi-do Korea; 2 Department of Ophthalmology, Institute of Vision Research, Yonsei University College of Medicine, Seoul, Korea; 3 Department of Ophthalmology, Asan Medical Center, University of Ulsan College of Medicine, 88, Olympic-Ro 43-Gil, Songpa-Gu, Seoul, 05505 South Korea; 4 Clinical Research Center, Asan Institute for Life Sciences, Asan Medical Center, University of Ulsan College of Medicine, Seoul, Korea; 5 Department of Clinical Epidemiology and Biostatistics, Asan Medical Center, University of Ulsan College of Medicine, Seoul, Korea

**Keywords:** Eye diseases, Medical research, Epidemiology

## Abstract

This population-based, retrospective cohort study aimed to evaluate the association between glaucoma surgery and all-cause and cause-specific mortality among Korean elderly patients with glaucoma. A total of 16210 elderly patients (aged ≥ 60 years) diagnosed with glaucoma between 2003 and 2012 were included, and their insurance data were analyzed. The participants were categorized into a glaucoma surgery cohort (n = 487), which included individuals who had diagnostic codes for open angle glaucoma (OAG) or angle closure glaucoma (ACG) and codes for glaucoma surgery, and a glaucoma diagnosis cohort (n = 15,723), which included patients who had codes for OAG and ACG but not for glaucoma surgery. Sociodemographic factors, Charlson Comorbidity Index score, and ocular comorbidities were included as covariates. Cox regression models were used to assess the association between glaucoma surgery and mortality. The incidence of all-cause mortality was 34.76/1,000 person-years and 27.88/1,000 person-years in the glaucoma surgery and diagnosis groups, respectively. The adjusted hazard ratio (HR) for all-cause mortality associated with glaucoma surgery was 1.31 (95% confidence interval [CI], 1.05–1.62, P = 0.014). The adjusted HR for mortality due to a neurologic cause was significant (HR = 2.66, 95% CI 1.18–6.00, P = 0.018). The adjusted HRs for mortality due to cancer (HR = 2.03, 95% CI 1.07–3.83, P = 0.029) and accident or trauma (HR = 4.00, 95% CI 1.55–10.34, P = 0.004) associated with glaucoma surgery for ACG were significant as well. Glaucoma surgery was associated with an increase of mortality in elderly patients with glaucoma. In particular, the risk of mortality associated with glaucoma surgery due to neurologic causes was significant.

## Introduction

Glaucoma is a leading cause of irreversible blindness and is expected to affect more than 100 million people worldwide by 2040^1,2^. Reduced vision due to glaucoma progression negatively affects a patient’s quality of life^3^. In a recent study of mortality and causes of death among the individuals with blindness in Korea^4^, the mortality risk recorded in the blindness group was 1.54 times higher than that of the control group. As glaucoma and visual impairment including blindness are closely related, an association between glaucoma and mortality is expected.

Using topical medications for the reduction of intraocular pressure (IOP) is considered the first line treatment for glaucoma. Surgical treatment is considered when the medical treatment does not sufficiently reduce IOP to decrease the rate of glaucomatous damage. Medical treatment is often combined with surgical treatment. However, since surgical treatment is used to induce maximum IOP reduction, the glaucoma status of patients who underwent glaucoma surgery is likely to differ from that of patients who only received medical treatment. Although previous studies have investigated the association between glaucoma and mortality^5–16^, none have studied the association between glaucoma surgery and mortality.

Therefore, we conducted this study to verify the association between glaucoma surgery and total and cause-specific mortality using the Korean National Health Insurance Service-Senior cohort (KNHIS-Senior), which is a nationwide cohort database that represents the entire elderly Korean population.

## Results

### Baseline characteristics

Table 1 shows the characteristics of patients with and without a history of glaucoma surgery. The study cohort included 16,210 patients with glaucoma (mean [SD] age, 72.6 [5.6] years). Among them, 487 patients underwent glaucoma surgery, and 15,723 patients did not undergo glaucoma surgery. Patients aged > 70 years tended not to opt for glaucoma surgery (absolute standardized difference, ASD = 0.228). Additionally, the individuals in the glaucoma surgery group were significantly younger than those in the glaucoma diagnosis group (ASD = 0.237). There were no significant differences between both groups regarding other variables except the ratio of patients who had diabetes mellitus (DM) with ophthalmic manifestations. A significantly greater ratio of patients having DM with ophthalmic manifestations were identified in the glaucoma surgery group than in the glaucoma diagnosis group (ASD = 0.135).

**Table 1:** Baseline characteristics of patients according to history of glaucoma surgery.

Variable	Total (n = 16,210)	Surgery group (n = 487)	Non-surgery group (n = 15,723)	ASD^a^
**Age (years)**				0.228
< 70	4862 (30.0)	192 (39.4)	4670 (29.7)	
70–74	5912 (36.5)	165 (33.9)	5747 (36.6)	
75–79	3509 (21.6)	89 (18.3)	3420 (21.8)	
80–84	1454 (9.0)	34 (7.0)	1420 (9.0)	
≥ 85	473 (2.9)	7 (1.4)	466 (3.0)	
Mean ± SD	72.6 ± 5.6	71.4 ± 5.4	72.7 ± 5.6	0.237
**Sex**				0.022
Male	7051 (43.5)	217 (44.6)	11,646 (40.0)	
Female	9159 (56.5)	270 (55.4)	17,498 (60.0)	
**Residence**				0.036
Metropolitan	7033 (43.4)	203 (41.7)	6830 (43.4)	
Provincial	9177 (56.6)	284 (58.3)	8893 (56.6)	
**Income**				0.050
Below 20 percentiles	2954 (18.2)	98 (20.1)	2856 (18.2)	
Above 20 percentiles	13,256 (81.8)	389 (79.9)	12,867 (81.8)	
**CCI**				0.077
0	1798 (11.1)	57 (11.7)	1741 (11.1)	
1	2742 (16.9)	82 (16.8)	2660 (16.9)	
2	2885 (17.8)	94 (19.3)	2791 (17.8)	
3	2519 (15.5)	77 (15.8)	2442 (15.5)	
4	2052 (12.7)	65 (13.3)	1987 (12.6)	
≥ 5	4214 (26.0)	112 (23.0)	4102 (26.1)	
BMI (kg/m^2^)	23.9 ± 3.2	23.9 ± 3.2	23.9 ± 3.2	0.019
**Smoking status**				0.060
Non-smoker	12,847 (79.3)	393 (80.7)	12,454 (79.2)	
Ex-smoker	1601 (9.9)	40 (8.2)	1561 (9.9)	
Current smoker	1762 (10.9)	54 (11.1)	1708 (10.9)	
**Alcohol consumption**				0.041
No	12,581 (77.6)	386 (79.3)	12,195 (77.6)	
Yes	3629 (22.4)	101 (20.7)	3528 (22.4)	
**Regular exercise**				0.028
No	13,381 (82.5)	407 (83.6)	12,974 (82.5)	
Yes	2829 (17.5)	80 (16.4)	2749 (17.5)	
**Age-related macular degeneration**				0.019
No	15,603 (96.3)	467 (95.9)	15,136 (96.3)	
Yes	607 (3.7)	20 (4.1)	587 (3.7)	
**DM with ophthalmic manifestation**s				0.135
No	15,609 (96.3)	455 (93.4)	15,154 (96.4)	
Yes	601 (3.7)	32 (6.6)	569 (3.6)	
**Severe cataract**				0.040
No	12,968 (80.0)	397 (81.5)	12,571 (80.0)	
Yes	3242 (20.0)	90 (18.5)	3152 (20.0)	

*BMI* body mass index, *CCI* Charlson Comorbidity Index, *DM* diabetes mellitus, *ASD* absolute standardized difference.

Data are expressed as mean ± standard deviation (SD) or n (%).

^a^ASD of > 0.1 is considered a meaningful imbalance.

### Incidence of mortality

Table 2 indicates the mortality rates of elderly Korean patients with or without a history of glaucoma surgery. The incidence of mortality by all causes was 34.76 deaths per 1000 person-years in the glaucoma surgery group and 27.88 deaths per 1000 person-years in the glaucoma diagnosis group. The incidence of mortality due to neurologic causes showed remarkable differences between groups. It was 2.51 deaths per 1000 person-years in the glaucoma surgery group and 0.93 deaths per 1000 person-years in the glaucoma diagnosis group.

**Table 2:** Mortality rates of Korean elderly patients according to history of glaucoma surgery.

Cause of mortality	Mortality rate, No. of deaths/total person-years (incidence per 1000 Person-years; 95% CI)	
	Surgery group	Non-surgery group
All-cause	83/2388 (34.76; 28.03–43.10)	2951/105,850 (27.88; 26.89–28.90)
Cancer	21/2388 (8.79; 5.73–13.49)	860/105,850 (8.12; 7.60–8.69)
Vascular	17/2388 (7.12; 4.43–11.45)	695/105,850 (6.57; 6.10–7.07)
Pulmonary	9/2388 (3.77; 1.96–7.24)	322/105,850 (3.04; 2.73–3.39)
Neurologic	6/2388 (2.51; 1.13–5.59)	98/105,850 (0.93; 0.76–1.13)
Infectious	2/2388 (0.84; 0.21–3.35)	86/105,850 (0.81; 0.66–1.00)
Accident or trauma	7/2388 (2.93; 1.40–6.15)	227/105,850 (2.14; 1.88–2.44)

Cox model with glaucoma surgery status as a time-varying covariate.

*CI* confidence interval.

Table 3 shows the hazard ratio (HR) for all-cause and cause-specific mortality in elderly patients with or without a history of glaucoma surgery. One unadjusted Cox model and three adjusted Cox models were identified in the analysis of the adjusted variables. Patients who had a history of glaucoma surgery were more prone to all-cause mortality than were those without a history of glaucoma surgery. The HRs for all-cause mortality in all the adjusted Cox models were significant (HR = 1.28, 95% confidence interval, CI 1.03–1.59, P = 0.023 in model 1; HR = 1.29, 95% CI 1.04–1.60, P = 0.02 in model 2; and HR = 1.31, 95% CI 1.05–1.62, P = 0.014 in model 3). Analysis of cause-specific HRs showed that only the HRs for mortality due to neurologic causes were significant in all Cox models. In the unadjusted Cox model, the HR was 2.3, with a 95% CI ranging from 1.02 to 5.22 (P = 0.045). Additionally, the HRs for mortality related to neurologic causes were 2.59 (95% CI 1.15–5.84, P = 0.022), 2.71 (95% CI 1.20–6.12, P = 0.017), and 2.66 (95% CI 1.18–6.00, P = 0.018) in Cox model 1, model 2, and model 3, respectively.

**Table 3:** Hazard ratios of total and cause-specific mortality in Korean elderly patients according to history of glaucoma surgery.

Cause of mortality (No. of participants)	Unadjusted cox model Hazard ratio (95% CI)^a^	*P*-value	Adjusted cox model Hazard Ratio (95% CI)^a^^,b^	*P*-value	Adjusted cox model Hazard ratio (95% CI)^a^^,c^	*P*-value	Adjusted cox model Hazard ratio (95% CI)^a^^,d^	*P*-value
All-cause	1.16 (0.93–1.43)	0.184	1.28 (1.03–1.59)	0.023	1.29 (1.04–1.60)	0.020	1.31 (1.05–1.62)	0.014
Cancer	1.06 (0.69–1.63)	0.792	1.14 (0.74–1.76)	0.541	1.16 (0.75–1.79)	0.505	1.17 (0.75–1.80)	0.487
Vascular	0.98 (0.61–1.58)	0.938	1.10 (0.68–1.78)	0.698	1.11 (0.68–1.79)	0.683	1.12 (0.69–1.80)	0.654
Pulmonary	1.09 (0.56–2.10)	0.805	1.25 (0.64–2.41)	0.514	1.22 (0.63–2.34)	0.555	1.26 (0.66–2.40)	0.488
Neurologic	2.30 (1.02–5.22)	0.045	2.59 (1.15–5.84)	0.022	2.71 (1.20–6.12)	0.017	2.66 (1.18–6.00)	0.018
Infectious	0.93 (0.23–3.80)	0.917	1.02 (0.25–4.17)	0.975	1.02 (0.25–4.22)	0.979	1.03 (0.25–4.26)	0.971
Accident or trauma	1.38 (0.65–2.91)	0.403	1.49 (0.71–3.14)	0.297	1.59 (0.75–3.36)	0.225	1.61 (0.77–3.39)	0.209

*CI* confidence interval, *DM* diabetes mellitus.

^a^Cox model with vitreoretinal surgery status as a time-varying covariate.

^b^Adjusted for age and sex.

^c^Adjusted for age, sex, income, region, Charlson Comorbidity Index score (0, 1, 2, 3, 4, ≥ 5), age-related macular degeneration, DM with ophthalmic manifestations, and cataract severity.

^d^Adjusted for age, sex, income, region, Charlson Comorbidity Index score (0, 1, 2, 3, 4, ≥ 5), age-related macular degeneration, DM with ophthalmic manifestations, cataract severity, smoking, drinking, exercise, and body mass index.

The HRs for the incidence of mortality related with glaucoma surgery after the adjustment of covariates are presented in Table 4. All variables except age 75 to 79 years, Charlson comorbidity index (CCI) score of 3, and an ex-smoker status showed HRs greater than 1. However, analysis of the interaction between these variables and the effect of glaucoma surgery on mortality showed no significant results.

**Table 4:** Hazard ratios of mortality in Korean elderly patients with glaucoma according to age, sex, residence, income, Charlson comorbidity index score, and ocular comorbidities.

	Adjusted hazard ratio (95% CI)	*P*-value	*P*-value for interaction
**Age (years)**			0.198
< 70	1.79 (1.27–2.53)	0.001	
70–74	1.17 (0.77–1.78)	0.455	
75–79	0.91 (0.58–1.45)	0.700	
80–84	1.22 (0.61–2.45)	0.580	
≥ 85	1.10 (0.47–2.55)	0.833	
**Sex**			0.096
Male	1.53 (1.17–2.00)	0.002	
Female	1.05 (0.73–1.50)	0.797	
**Residence**			0.233
Metropolitan	1.55 (1.09–2.21)	0.015	
Provincial	1.18 (0.90–1.55)	0.219	
**Income**			0.260
Below 20 percentiles	1.63 (1.06–2.50)	0.025	
Above 20 percentiles	1.23 (0.96–1.57)	0.102	
**CCI**			0.214
0	1.49 (0.84–2.62)	0.172	
1	1.30 (0.73–2.33)	0.377	
2	1.71 (1.02–2.89)	0.043	
3	0.43 (0.16–1.12)	0.084	
4	1.16 (0.61–2.18)	0.654	
≥ 5	1.53 (1.08–2.16)	0.016	
**Age-related macular degeneration**			0.669
No	1.32 (1.06–1.64)	0.013	
Yes	1.01 (0.30–3.40)	0.993	
**DM with ophthalmic manifestations**			0.299
No	1.25 (0.99–1.58)	0.059	
Yes	1.76 (0.96–3.20)	0.066	
**Severe cataract**			
No	1.33 (1.05–1.70)	0.019	0.713
Yes	1.21 (0.77–1.91)	0.415	
**Smoking status**			0.585
Non-smoker	1.35 (1.06–1.71)	0.016	
Ex-smoker	0.84 (0.34–2.06)	0.697	
Current smoker	1.41 (0.82–2.41)	0.210	
**Alcohol consumption**			0.834
No	1.32 (1.03–1.69)	0.027	
Yes	1.26 (0.82–1.92)	0.293	
**Regular exercise**			0.090
No	1.21 (0.96–1.53)	0.114	
Yes	1.98 (1.18–3.32)	0.010	
**BMI**			0.542
< 23	1.49 (1.10–2.02)	0.010	
23–24.9	1.27 (0.80–1.99)	0.308	
≥ 25	1.12 (0.75–1.69)	0.571	

Glaucoma diagnosis group was used as a reference in all models. Adjusted for age, sex, income, region, Charlson Comorbidity Index score (0, 1, 2, 3, 4, and ≥ 5), age-related macular degeneration, DM with ophthalmic manifestations, cataract severity, smoking, drinking, exercise, and BMI.

*BMI* body mass index, *CCI* Charlson Comorbidity Index, *DM* diabetes mellitus.

We investigated the HRs for cause-specific mortality according to the history of glaucoma surgery for open angle glaucoma (OAG) and angle closure glaucoma (ACG) (Table 5). The baseline characteristics of patients who had OAG and ACG are presented in Supplementary Tables S1 and S2. For OAG, there were no significant HRs for cause-specific mortality related with glaucoma surgery in all unadjusted and adjusted analyses. However, for ACG, the HRs for total mortality and mortality caused by accident or trauma were significant in the unadjusted and adjusted analyses. After adjusting for all variables (model 3), the HR of all-cause mortality and mortality caused by accident or trauma was 1.82 (95% CI 1.27–2.62, P = 0.001) and 4.00 (95% CI 1.55–10.34, P = 0.004), respectively. Additionally, increase of cancer mortality was significantly associated with glaucoma surgery for ACG. Regarding the results of the adjusted Cox models, the HR of cancer-related mortality was 1.99 (95% CI 1.07–3.71, P = 0.03), 2.03 (95% CI 1.08–3.84, P = 0.028), and 2.03 (95% CI 1.07–3.83, P = 0.029) in models 1, 2, and 3, respectively.

**Table 5:** Hazard ratios of total and cause-specific mortality in Korean elderly patients with open-angle glaucoma and angle closure glaucoma according to history of glaucoma surgery.

Cause of mortality (No. of participants)	Unadjusted cox model Hazard ratio (95% CI)^a^	*P*-value	Adjusted cox model Hazard ratio (95% CI)^a^^,b^	*P*-value	Adjusted cox model Hazard ratio (95% CI)^a^^,c^	*P*-value	Adjusted cox model Hazard ratio (95% CI)^a^^,d^	*P*-value
**Open-angle glaucoma**								
All-cause	1.03 (0.79–1.35)	0.816	1.10 (0.84–1.44)	0.482	1.10 (0.84–1.43)	0.498	1.12 (0.86–1.46)	0.413
Cancer	0.79 (0.43–1.43)	0.434	0.81 (0.45–1.48)	0.496	0.81 (0.45–1.48)	0.499	0.82 (0.45–1.50)	0.522
Vascular	0.96 (0.54–1.71)	0.894	1.05 (0.59–1.86)	0.878	1.04 (0.59–1.85)	0.884	1.07 (0.60–1.89)	0.828
Pulmonary	1.15 (0.55–2.41)	0.714	1.24 (0.59–2.62)	0.570	1.21 (0.58–2.52)	0.603	1.27 (0.62–2.62)	0.519
Neurologic	2.14 (0.80–5.75)	0.132	2.37 (0.89–6.33)	0.085	2.47 (0.93–6.57)	0.071	2.41 (0.91–6.39)	0.078
Infectious	1.35 (0.33–5.57)	0.677	1.45 (0.35–5.93)	0.608	1.44 (0.34–5.99)	0.619	1.46 (0.35–6.12)	0.604
Accident or trauma	0.56 (0.14–2.27)	0.418	0.58 (0.15–2.31)	0.442	0.62 (0.16–2.46)	0.496	0.63 (0.16–2.51)	0.516
**Angle closure glaucoma**								
All-cause	1.57 (1.09–2.25)	0.015	1.80 (1.26–2.58)	0.001	1.83 (1.26–2.64)	0.001	1.82 (1.27–2.62)	0.001
Cancer	1.76 (0.93–3.34)	0.081	1.99 (1.07–3.71)	0.030	2.03 (1.08–3.84)	0.028	2.03 (1.07–3.83)	0.029
Vascular	1.11 (0.46–2.67)	0.822	1.27 (0.52–3.09)	0.595	1.27 (0.53–3.06)	0.593	1.25 (0.52–2.98)	0.616
Pulmonary	1.05 (0.25–4.35)	0.949	1.32 (0.32–5.44)	0.702	1.24 (0.29–5.28)	0.768	1.25 (0.30–5.27)	0.762
Neurologic	2.78 (0.63–12.27)	0.176	3.10 (0.70–13.76)	0.137	3.22 (0.71–14.58)	0.129	3.22 (0.71–14.62)	0.130
Infectious	–	–	–	–	–	–	–	–
Accident or trauma	3.36 (1.31–8.61)	0.011	3.78 (1.47–9.71)	0.006	4.04 (1.57–10.41)	0.004	4.00 (1.55–10.34)	0.004

*DM* diabetes mellitus.

^a^Cox model with vitreoretinal surgery status as a time-varying covariate.

^b^Adjusted for age and sex.

^c^Adjusted for age, sex, income, region, Charlson Comorbidity Index (0, 1, 2, 3, 4, and ≥ 5), age-related macular degeneration, DM with ophthalmic manifestations, and cataract severity.

^d^Adjusted for age, sex, income, region, Charlson Comorbidity Index (0, 1, 2, 3, 4, and ≥ 5), age-related macular degeneration, DM with ophthalmic manifestations, cataract severity, smoking, drinking, exercise, and body mass index.

## Discussion

In the present study of patients with OAG and ACG, the glaucoma surgery group had a higher mortality rate than that of the glaucoma diagnosis group, regardless of the cause of mortality. Additionally, after adjusting for variables including demographic factors and systemic and ocular comorbidities, the hazard of all-cause mortality in elderly patients who underwent glaucoma surgery increased by about 30% compared with that of patients who did not undergo glaucoma surgery. Moreover, the risk of mortality due to neurologic causes was significantly higher in elderly patients who underwent glaucoma surgery than in those who did not.

The association between glaucoma and neurologic disease has been investigated in several previous studies by evaluating pathogenic mechanisms like neurotoxicity caused by amyloid-beta, Tau protein, autophagy downregulation, dopamine depletion, and alteration of the hemodynamics of a cerebral artery with white matter lesions^17^. Several experts have suggested a correlation between glaucoma and stroke^18–21^. Alzheimer’s disease^22–24^ and Parkinson’s disease^25–27^ are representative neurologic disorders that are reportedly associated with glaucoma. In studies using brain magnetic resonance imaging, structural and functional alterations were observed in patients with glaucoma^25–27^. These results support the hypothesis that glaucoma should be considered a central nervous system disorder. In the present study, the incidence of mortality due to neurologic causes was noticeably different between the glaucoma surgery group (2.51 per 1000 person-years) and the glaucoma diagnosis group (0.93 per 1000 person-years). Additionally, glaucoma surgery was significantly associated with a > 2.5 time increase of HR for mortality due to neurologic causes, even after all variables were adjusted. These results show the importance of assessing patients, especially those with OAG or ACG who have undergone glaucoma surgery, for neurological disorders.

The analysis of HRs for mortality related with glaucoma surgery, which were stratified according to covariates, showed that almost all HRs were > 1 (Table 3). In particular, the HRs adjusted for age below 70 years, male, residing in a metropolitan area, income below the 20th percentile, a CCI score of 2 and ≥ 5, no age-related macular degeneration, no severe cataract, non-smoking, no alcohol consumption, regular exercise, and body mass index (BMI) under 23 kg/m^2^ were significant. These are conditions thought to decrease the morbidity of ocular or systemic diseases. Since there were no significant p-values for interaction even though there were significant adjusted HRs, further studies are needed to determine the role of these conditions in increasing the risk of mortality in patients who have undergone glaucoma surgery. However, the results of the present study show that the hazard of mortality related with glaucoma surgery can increase even if conditions that increase the possibility of ocular or systemic disease are absent. This enhances the reliability of our findings, which confirm the link between glaucoma surgery and mortality, thereby serving as important evidence regarding the relationship between glaucoma and mortality.

We investigated the cause-specific hazard of mortality by analyzing OAG and ACG separately. Interestingly, these two types of glaucoma were associated with increased hazard for mortality of different causes. Patients with OAG showed an increased risk of death due to all, vascular, pulmonary, neurologic, and infectious causes; however, these were not significant. On the other hand, patients with ACG showed an increased risk of mortality due to all causes. In particular, glaucoma surgery was significantly associated with an increase in the risk for all-cause mortality, mortality caused by cancer, and accident or trauma-related mortality in patients with ACG, who are known to have an approximately three-fold increased risk of having severe binocular vision loss compared to patients with OAG^28,29^. In a recent meta-analysis, the pooled relative risk of mortality due to visual impairment was 1.36^30^. In another national sample cohort study, the blindness group showed a 1.54 times increased risk of mortality compared with the control group^4^. Additionally, another study, in which the Beijing Eye Study was evaluated, suggested the presence of a strong association between mortality and primary ACG but not between mortality and primary OAG^12^. Therefore, it could be considered that the results of the present study, demonstrating a remarkable increase in the risk of mortality related with glaucoma surgery for ACG, are reliable.

Adjusted HR for cancer-related mortality associated with glaucoma surgery for ACG was 2.03 (P = 0.029). A recent study using national insurance data to identify the risk of cancer in patients with glaucoma showed that the adjusted HR for cancer was 1.049 with varying HRs for different types of cancer^31^. Chronic inflammation and environmental factors such as smoking are thought to influence the relationship between cancer and glaucoma. However, the risks of cancer development in patients with OAG or ACG were not assessed in previous studies. We found conflicting results in the analysis of the effects of glaucoma surgery on the risk of cancer-related death in patients with OAG and ACG. Thus, if researchers wish to conduct further studies on the association between glaucoma, cancer, and mortality, classifying the types of glaucoma is necessary.

In the present study, risk of mortality due to accident or trauma was significantly increased fourfold by glaucoma surgery for ACG. However, with OAG, the HR of mortality caused by accident or trauma was 0.63 and was not significant. Restrictions in daily activities such as walking, balancing, and driving not only reduce the quality of life but also increase mortality in patients with glaucoma^32^. Several studies have shown that visual impairment related with glaucomatous damage is associated with motor vehicle collisions and falls, which are major causes of death related to unintended injury^33–39^. Since our study was conducted using claims data, there were limitations in accurately evaluating the visual functions of the patients. Additionally, since severity of impaired visual function, whether binocular or monocular, and location of visual field defects are important factors in evaluating the effect of visual functional damage on the quality of life of patients with glaucoma regardless of angle status, further studies are required to determine the reasons for the obvious differences between the hazards of cause-specific mortality associated with glaucoma surgery for the two types of glaucoma. However, considering the characteristics of ACG, which has a higher possibility of causing serious decline in visual function than does OAG, the possibility of progressed glaucomatous impairment of visual function, which is implied by a history of glaucoma surgery, and the fact that the risk of trauma-related death increases with age, our finding regarding trauma and accident-related mortality can be considered reliable and useful for the follow-up of patients aged over 60 years who underwent surgery for ACG. Furthermore, the significance of the findings of our study reflects the fact that deaths associated with trauma and accident can be sufficiently prevented by the efforts of patients and doctors.

This study has several limitations. First, it was a retrospective study. Second, since we used national medical claims data, clinical information on disease diagnosis or severity was insufficient. Other factors that could potentially affect the transition from medical treatment to surgical treatment could not be included in our analyses. In addition, only patients who visited medical facilities were included in this study. Third, this study was conducted on patients aged ≥ 60 years with OAG and ACG; this should be considered when applying our results regarding the association of glaucoma surgery and increased risk of mortality to clinical practice. Finally, although we used a large database, the population consisted of mostly Koreans. Considering that Asians account for about 77% of the world’s primary ACG cases, the racial restrictions of the study population may affect the results of our subgroup analysis according to the type of glaucoma.

In summary, we demonstrated that glaucoma surgery was significantly associated with an increase in mortality in elderly Korean patients with OAG and ACG. The adjusted HR for mortality related with glaucoma surgery increased by about 30%. The results of our study showed that the risk of mortality due to neurologic causes was significantly increased, 2.66-fold. The increase in the risk of mortality due to neurologic causes was marginal in patients with OAG, whereas cancer and accident or trauma-related mortality risks were significantly increased in patients with ACG. These suggest that continuous surveillance by healthcare providers and patients during follow-up is necessary for the reduction and prevention of deaths of patients who undergo glaucoma surgery. Additionally, we believe that it is important to detect and treat glaucoma early to minimize the need for surgical treatment.

## Methods

### Study design

This was a population-based retrospective cohort study conducted using the KNHIS-Senior database. The health insurance system in South Korea is a nationwide universal single payer system managed by the KNHIS. The KNHIS-Senior database is one of the databases provided by the KNHIS. It includes the data of 558,147 individuals randomly sampled from 10% of the approximately 5.5 million South Korean population aged > 60 years and includes information on age, sex, general health examinations, hospital and pharmacy visits, disease diagnoses, status, procedures, and prescribed medications. The general health examination database includes the results of the health screening examination conducted by the KNHIS^40^.

The baseline information of the individuals was collected during health screening examinations. Data on smoking habits, alcohol habits, and regular exercise were collected using self-administered questionnaires. Height, weight, and blood pressure were measured, and BMI was calculated as weight in kilograms divided by height in meters squared.

The KNHIS uses the Korean Electronic Data Interchange (KEDI) codes and the Korean Standard Classification of Diseases (KCD) codes, a system similar to the International Classification of Diseases (ICD). This study was conducted according to the ethical principles outlined in the Declaration of Helsinki. Informed consent was not obtained because anonymized and de-identified information was used for analyses. As KNHIS-Senior database comprises publicly opened data, the Institutional Review Board of Asan Medical Center and the University of Ulsan College of Medicine approved a waiver for reviewing this study (2020-1197).

### Study population

We selected the study population from those included in the KNHIS-Senior database between January 1, 2002, and December 31, 2015. To minimize the impact of surveillance bias, we set a wash-out period between January 1, 2002, and December 31, 2002. The inclusion criterion was the presence of at least one NHIS record with the following conditions: a KCD-7 code for OAG (H401) or ACG (H402) between January 1, 2003, and December 31, 2012, and glaucoma surgery between January 1, 2003, and December 30, 2015 (Table 6). The eligible individuals were classified into a glaucoma surgery group, which consisted of patients with ICD-10 diagnosis codes for OAG or ACG and KEDI codes for glaucoma surgeries, and a glaucoma diagnosis group, which included people with glaucoma diagnosis codes, but without a KEDI code for glaucoma surgeries. Patients with age less than 60 years and codes for secondary glaucoma were excluded.

**Table 6:** Korean electronic data interchange codes for glaucoma surgery.

KEDI code	Classification
S5033	Trabeculectomy
S5038	Stent insertion in sub-conjunctiva
S5039	Stent insertion in Schlemm’s cannal
S5040	Nonpenetrating filtration surgery (Deep sclerectomy, Viscocanalostomy)
S5042	Filtration operation (sclerotomy, sclerectomy,
S5043	Trabeculectomy with laser
S5044	Photocoagulation for iris, Ciliary body
S5045	Cyclocryotherapy
S5047	Trabeculotomy under microscopy
S5048	Schlemm's canal opening under microscopy
S5049	Glaucoma implant surgery
SZ670	Glaucoma aqueous tube insertion

*KEDI* Korean Standard Classification of Disease.

### Exposure and outcome

The exposure of interest was glaucoma surgery. Both the glaucoma surgery and glaucoma diagnosis groups were followed up from the date of the initial diagnosis of glaucoma. The primary outcome of interest was total and cause-specific mortality at any point from the time of patient inclusion in the study until the end of the study on December 31, 2015. Mortality status was confirmed using an indicator variable in the KNHIS-Senior database, which contains information on total and cause-specific mortality. Causes of death were grouped into cancer, vascular, pulmonary, neurologic, infectious, accident or trauma-related conditions (Supplementary Table S3).

For the glaucoma surgery group, the time between glaucoma diagnosis and surgery was defined as the follow-up period for glaucoma diagnosis, and the time after surgery was defined as the follow-up period for glaucoma surgery^41^. For the glaucoma diagnosis group, time until death was calculated as the number of days from glaucoma diagnosis to death^41^. Individuals with no death record in the database were censored on the last known date or on December 31, 2015, if still enrolled.

### Covariates

Information on sociodemographic factors, including age at the time of glaucoma diagnosis, sex, residence (metropolitan areas, including Seoul and large cities, and provincial regions, including small cities and rural areas), household income level (categorized below or above 20% of median income), smoking and alcohol habits, regular exercise, and BMI, were obtained from the database. The CCI score^42,43^ was used as a covariate to represent overall general health status for matching between two groups. It is a weighted index of systemic disease burden according to the presence or absence of 17 systemic diseases. The higher the CCI score, the higher the systemic disease burden. Individuals with a CCI score of 0 to 6 points, which can be used to predict the risk of one-year mortality, were classified according to their systemic disease profiles. Ocular comorbidities such as age-related macular degeneration, DM with ophthalmic manifestations, and severe cataract were also used as matching variables. KCD-7 codes for systemic and ocular comorbidities are presented in Supplementary Table S4.

### Statistical analysis

Patient characteristics and mortality rates were descriptively compared according to glaucoma surgery status. Cox regression models with glaucoma surgery status (exposure) as time-varying covariates were used to obtain covariate-adjusted associations between glaucoma surgery and time until death due to any cause and between glaucoma surgery and time until death attributed to cancer, vascular, pulmonary, neurologic, infectious, accident or trauma-related conditions. HR and 95% CIs were calculated. Three models with increasing degrees of adjustment to account for potential confounding factors at baseline were used. Model 1 was adjusted for age (< 70, 70–74, 75–79, 80–84, and ≥ 85 years) and sex. Model 2 was additionally adjusted for income level, region, CCI, age-related macular degeneration, DM with ophthalmic manifestations, and severe cataract. In model 3, smoking status (non-smoker, ex-smoker, current smoker), alcohol intake (no, yes), exercise (no, yes), and BMI (continuous) were additionally adjusted.

All statistical analyses were conducted using the Statistical Analysis System (SAS) software version 9.4 (SAS Institute, Cary, NC, United States), and a two-sided P value of < 0.05 was considered statistically significant. To compare the baseline characteristics between the groups, ASD, calculated as the difference between means or proportions divided by a pooled estimate of the standard deviation, was used. Unlike the traditional methods used to test for significance, ASD is insensitive to sample size. Therefore, it is useful for identifying meaningful differences. An ASD value of > 0.1 was considered clinically significant^44^.

## Supplementary Information

Supplementary Information.

## Data Availability

The data that support the findings of this study are available from NHIS, but restrictions apply to the availability of these data, which were used under license for the current study; therefore, these are not publicly available. Data are, however, available from the authors upon reasonable request and with permission of NHIS.
