# Publisher Correction: Association between glaucoma surgery and all-cause and cause-specific mortality among elderly patients with glaucoma: a nationwide population-based cohort study

**DOI:** 10.1038/s41598-021-97817-z

**Published:** 2021-09-07

**Authors:** Sang Yeop Lee, Hun Lee, Ji Sung Lee, Sol Ah Han, Yoon Jeon Kim, Jae Yong Kim, Hungwon Tchah

**Affiliations:** 1 Department of Ophthalmology, Yongin Severance Hospital, Yonsei University College of Medicine, Yongin, Gyeonggi‑do Korea; 2 Department of Ophthalmology, Institute of Vision Research, Yonsei University College of Medicine, Seoul, Korea; 3 Department of Ophthalmology, Asan Medical Center, University of Ulsan College of Medicine, 88, Olympic‑Ro 43‑Gil, Songpa‑Gu, Seoul, 05505 South Korea; 4 Clinical Research Center, Asan Institute for Life Sciences, Asan Medical Center, University of Ulsan College of Medicine, Seoul, Korea; 5 Department of Clinical Epidemiology and Biostatistics, Asan Medical Center, University of Ulsan College of Medicine, Seoul, Korea

Correction to: *Scientific Reports* https://doi.org/10.1038/s41598-021-96063-7, published online 23 August 2021

The Funding section in the original version of this Article was omitted. The Funding section now reads:

“This research was supported by Research and Business Development Program through the Korea Institute for Advancement of Technology (KIAT) funded by the Ministry of Trade, Industry and Energy (MOTIE) (grant number: P0014063), by the Korea Medical Device Development Fund grant funded by the Korea government (the Ministry of Science and ICT, the Ministry of Trade, Industry and Energy, the Ministry of Health & Welfare, the Ministry of Food and Drug Safety) (Project Number: 9991006821, KMDF_PR_20200901_0148), and by a grant from the Asan Institute for Life Sciences, Asan Medical Center, Seoul, Korea (2021IP0061-1). The funding agency had no role in the design or conduct of this study; collection, management, analysis, or interpretation of the data; preparation, review, or approval of the manuscript; or in the decision to submit the manuscript for publication.”

The original Article has been corrected.

