# Supplementary material for: Association between glaucoma surgery and all-cause and cause-specific mortality among elderly patients with glaucoma: a nationwide population-based cohort study
Source: Sci Rep. 2021 Aug 23;11:17055. doi: 10.1038/s41598-021-96063-7 (PMC8382742; doi:10.1038/s41598-021-96063-7)
Supplement: Supplementary file 1 — Supplementary Information. [file 41598_2021_96063_MOESM1_ESM.docx]

**Association between glaucoma surgery and all-cause and cause-specific mortality among elderly patients with glaucoma: A nationwide population-based cohort study**

Sang Yeop Lee, MD, Hun Lee, MD, PhD, Ji Sung Lee, PhD, Sol Ah Han, MD, Yoon Jeon Kim, MD, PhD, Jae Yong Kim, MD, PhD, Hungwon Tchah, MD, PhD

**Supplementary Table S1**. Baseline characteristics of patients according to history of glaucoma surgery for open angle glaucoma

|  | Glaucoma Diagnosis Group | Glaucoma surgery = Yes | Glaucoma surgery = No | P-value | ASD |
| --- | --- | --- | --- | --- | --- |
| N | 13996 | 363 | 13633 |  |  |
| Age (years) |  |  |  | <.0001 | 0.2650 |
| 61-69 | 4126 (29.5) | 148 (40.8) | 3978 (29.2) |  |  |
| 70-74 | 5137 (36.7) | 120 (33.1) | 5017 (36.8) |  |  |
| 75-79 | 3042 (21.7) | 61 (16.8) | 2981 (21.9) |  |  |
| 80-84 | 1281 (9.2) | 29 (8.0) | 1252 (9.2) |  |  |
| ≥85 | 410 (2.9) | 5 (1.4) | 405 (3.0) |  |  |
| Mean±SD | 72.7±5.5 | 71.3±5.5 | 72.7±5.5 | <.0001 | 0.2566 |
| Sex |  |  |  | 0.1465 | 0.0771 |
| Male | 6339 (45.3) | 178 (49.0) | 6161 (45.2) |  |  |
| Female | 7657 (54.7) | 185 (51.0) | 7472 (54.8) |  |  |
| Region |  |  |  | 0.9964 | 0.0002 |
| Metropolitan | 6055 (43.3) | 157 (43.3) | 5898 (43.3) |  |  |
| Provincial | 7941 (56.7) | 206 (56.7) | 7735 (56.7) |  |  |
| Income |  |  |  | 0.4866 | 0.0365 |
| Below 20 percentile | 2543 (18.2) | 71 (19.6) | 2472 (18.1) |  |  |
| Above 20 percentile high income | 11453 (81.8) | 292 (80.4) | 11161 (81.9) |  |  |
| BMI (kg/m2) | 23.9±3.2 | 24.1±3.0 | 23.9±3.2 | 0.3621 | 0.0498 |
| Smoking status |  |  |  | 0.6170 | 0.0540 |
| Non-smoker | 11012 (78.7) | 290 (79.9) | 10722 (78.6) |  |  |
| Ex-smoker | 1450 (10.4) | 32 (8.8) | 1418 (10.4) |  |  |
| Current smoker | 1534 (11.0) | 41 (11.3) | 1493 (11.0) |  |  |
| Alcohol consumption |  |  |  | 0.8805 | 0.0080 |
| No | 10803 (77.2) | 279 (76.9) | 10524 (77.2) |  |  |
| Yes | 3193 (22.8) | 84 (23.1) | 3109 (22.8) |  |  |
| Regular exercise |  |  |  | 0.6900 | 0.0210 |
| No | 11523 (82.3) | 296 (81.5) | 11227 (82.4) |  |  |
| Yes | 2473 (17.7) | 67 (18.5) | 2406 (17.6) |  |  |
| CCI |  |  |  | 0.8161 | 0.0798 |
| 0 | 1508 (10.8) | 42 (11.6) | 1466 (10.8) |  |  |
| 1 | 2307 (16.5) | 59 (16.3) | 2248 (16.5) |  |  |
| 2 | 2493 (17.8) | 72 (19.8) | 2421 (17.8) |  |  |
| 3 | 2184 (15.6) | 58 (16.0) | 2126 (15.6) |  |  |
| 4 | 1790 (12.8) | 40 (11.0) | 1750 (12.8) |  |  |
| ≥5 | 3714 (26.5) | 92 (25.3) | 3622 (26.6) |  |  |
| Age-Related Macular Degeneration |  |  |  | 0.8462 | 0.0102 |
| No | 13445 (96.1) | 348 (95.9) | 13097 (96.1) |  |  |
| Yes | 551 (3.9) | 15 (4.1) | 536 (3.9) |  |  |
| DM with Ophthalmic Manifestations |  |  |  | 0.0024 | 0.1387 |
| No | 13456 (96.1) | 338 (93.1) | 13118 (96.2) |  |  |
| Yes | 540 (3.9) | 25 (6.9) | 515 (3.8) |  |  |
| Severe cataract |  |  |  | 0.5007 | 0.0363 |
| No | 11218 (80.2) | 296 (81.5) | 10922 (80.1) |  |  |
| Yes | 2778 (19.8) | 67 (18.5) | 2711 (19.9) |  |  |

Data are expressed as the mean±SD, or n (%).

P-value from Student's t-test and Chi-square test

ASD, absolute standardized difference; BMI, body mass index; CCI, Charlson Comorbidity Index; DM, diabetes mellitus; SD, standard deviation

*ASD of >0.1 is considered a meaningful imbalance.

**Supplementary Table S2**. Baseline characteristics of patients according to history of glaucoma surgery for angle closure glaucoma

|  | Glaucoma Diagnosis Group | Glaucoma surgery = Yes | Glaucoma surgery = No | P-value | ASD |
| --- | --- | --- | --- | --- | --- |
| N | 2214 | 124 | 2090 |  |  |
| Age (years) |  |  |  | 0.4773 | 0.1944 |
| 61-69 | 736 (33.2) | 44 (35.5) | 692 (33.1) |  |  |
| 70-74 | 775 (35.0) | 45 (36.3) | 730 (34.9) |  |  |
| 75-79 | 467 (21.1) | 28 (22.6) | 439 (21.0) |  |  |
| 80-84 | 173 (7.8) | 5 (4.0) | 168 (8.0) |  |  |
| ≥85 | 63 (2.8) | 2 (1.6) | 61 (2.9) |  |  |
| Mean±SD | 72.2±5.6 | 71.5±5.0 | 72.3±5.7 | 0.1692 | 0.1341 |
| Sex |  |  |  | 0.8622 | 0.0161 |
| Male | 712 (32.2) | 39 (31.5) | 673 (32.2) |  |  |
| Female | 1502 (67.8) | 85 (68.5) | 1417 (67.8) |  |  |
| Region |  |  |  | 0.1024 | 0.1530 |
| Metropolitan | 978 (44.2) | 46 (37.1) | 932 (44.6) |  |  |
| Provincial | 1236 (55.8) | 78 (62.9) | 1158 (55.4) |  |  |
| Income |  |  |  | 0.3440 | 0.0850 |
| Below 20 percentile | 411 (18.6) | 27 (21.8) | 384 (18.4) |  |  |
| Above 20 percentile high income | 1803 (81.4) | 97 (78.2) | 1706 (81.6) |  |  |
| BMI (kg/m2) | 23.7±3.2 | 23.6±3.6 | 23.8±3.2 | 0.6405 | 0.0410 |
| Smoking status |  |  |  | 0.9846 | 0.0165 |
| Non-smoker | 1835 (82.9) | 103 (83.1) | 1732 (82.9) |  |  |
| Ex-smoker | 151 (6.8) | 8 (6.5) | 143 (6.8) |  |  |
| Current smoker | 228 (10.3) | 13 (10.5) | 215 (10.3) |  |  |
| Alcohol consumption |  |  |  | 0.0846 | 0.1698 |
| No | 1778 (80.3) | 107 (86.3) | 1671 (80.0) |  |  |
| Yes | 436 (19.7) | 17 (13.7) | 419 (20.0) |  |  |
| Regular exercise |  |  |  | 0.0808 | 0.1744 |
| No | 1858 (83.9) | 111 (89.5) | 1747 (83.6) |  |  |
| Yes | 356 (16.1) | 13 (10.5) | 343 (16.4) |  |  |
| CCI |  |  |  | 0.0670 | 0.2764 |
| 0 | 290 (13.1) | 15 (12.1) | 275 (13.2) |  |  |
| 1 | 435 (19.6) | 23 (18.5) | 412 (19.7) |  |  |
| 2 | 392 (17.7) | 22 (17.7) | 370 (17.7) |  |  |
| 3 | 335 (15.1) | 19 (15.3) | 316 (15.1) |  |  |
| 4 | 262 (11.8) | 25 (20.2) | 237 (11.3) |  |  |
| ≥5 | 500 (22.6) | 20 (16.1) | 480 (23.0) |  |  |
| Age-Related Macular Degeneration |  |  |  | 0.2396 | 0.0901 |
| No | 2158 (97.5) | 119 (96.0) | 2039 (97.6) |  |  |
| Yes | 56 (2.5) | 5 (4.0) | 51 (2.4) |  |  |
| DM with Ophthalmic Manifestations |  |  |  | 0.0794 | 0.1546 |
| No | 2153 (97.2) | 117 (94.4) | 2036 (97.4) |  |  |
| Yes | 61 (2.8) | 7 (5.6) | 54 (2.6) |  |  |
| Severe cataract |  |  |  | 0.4975 | 0.0640 |
| No | 1750 (79.0) | 101 (81.5) | 1649 (78.9) |  |  |
| Yes | 464 (21.0) | 23 (18.5) | 441 (21.1) |  |  |

Data are expressed as the mean ± SD, or n (%).

P-value from Student's t-test and Chi-square test

ASD, absolute standardized difference; BMI, body mass index; CCI, Charlson Comorbidity Index; DM, diabetes mellitus; SD, standard deviation

*ASD of >0.1 is considered a meaningful imbalance.

**Supplementary Table S3.** Korean Standard Classification of Diseases codes for mortality attributed to specific systemic condition

| **Type of Mortality** | **Eligible KCD Diagnosis Code** |
| --- | --- |
| Cancer death | C00-C14, C15-C26, C30-C39, C40-C41, C43-C44, C45-C49. C50, C51-C58, C60-C63, C64-C68, C69-C72, C73-C75, C76-C80, C81-C96, C97-C97, D00-D09, D10-D36, D37-D48 |
| Vascular death | I00-I02, I05-I09, I10-I15, I20-I25, I26-I28, I30-I52, I60-I69, I70-I79, I80-I89, I95-I99 |
| Pulmonary death | J00-J06, J09-J18, J20-J22, J30-J39, J40-J47, J60-J70, J80-J84, J85-J86, J90-J94, J95-J99 |
| Neurologic death | G00-G09, G10-G14, G20-G26, G30-G32, G35-G37, G40-G47, G50-G59, G60-G64, G70-G73, G80-G83, G90-G99 |
| Infection-related death | A00-A09, A15-A19, A20-A28, A30-A49, A50-A64, A65-A69, A70-A74, A75-A79, A80-A89, A90-A99, B00-B09, B15-B19, B20-B24, B25-B34, B35-B49, B50-B64, B65-B83, B85-B89, B90-B94, B95-B98, B99 |
| Accident or trauma-related death | V01-V99, W00-W99, X00-X84, X85-Y09, Y10-Y34, Y35-Y36, Y40-Y59, Y60-Y69, Y70-Y82, Y83-Y84, Y85-Y89, Y90-Y98 |

KCD, Korean Standard Classification of Diseases.

**Supplementary Table S4.** Korean Standard Classification of Diseases codes for comorbidities

| **Comorbidity** | **Eligible KCD Diagnosis Code** |
| --- | --- |
| Lymphomas | C81-C86, C88 |
| Multiple myeloma/leukemia | C90-C96 |
| Malignant neoplasms | C00-97 |
| Myocardial infarction | I21-I23 |
| Heart failure | I50, I97.1, I09.9, I11.0, I13.0, I13.2 |
| Cerebrovascular disease | I60-I69 |
| Peripheral vascular disease | I73 |
| Chronic pulmonary disease | J44, I27.9, J84 |
| Cirrhosis | K70.3, K71.7, K74, K76.1 |
| Hepatic failure | K70.4, K71.1, K72 |
| AIDS | B20-B24 |
| Hemi/paraplegia | G04.1, G11.4, G81-82, I69.006, I69.106, I69.206, I69.306, I69.406 |
| Rheumatologic disease | M05, M06, M10, M12.0, M12.3, M30-M36 |
| Dementia | F00-F03, F05.1, G31.82 |
| Diabetes mellitus | E10-E14 |
| Diabetes mellitus with complications | E10.0-E10.8, E11.0-E11.8, E12.0-E12.8, E13.0-E13.8, E14.0-E14.8 |
| Chronic renal disease | E10.22, E12.22, E13.22, E14.22, I12, I13 |
| Peptic ulcer disease | K27 |
| Age-related macular degeneration | H35.30, H35.31, H35.39 |
| Diabetes mellitus with ophthalmic manifestations | E10.3^†^, E11.3^†^, E12.3^†^, E13.3^†^, E14.3^†^ |

KCD, Korean Standard Classification of Diseases; AIDS, acquired immune deficiency syndrome.

^†^1,2,3,4,8.
